# Inferior plant competitor allocates more biomass to belowground as a result of greater competition for resources in heterogeneous habitats

**DOI:** 10.3389/fpls.2023.1184618

**Published:** 2023-09-19

**Authors:** Jian Zhou, Ziwen Ma, Yuehui Jia, Jie Liu, Yuping Yang, Wei Li, Lijuan Cui

**Affiliations:** ^1^ College of Bioscience and Resource Environment, Beijing University of Agriculture, Beijing, China; ^2^ Key laboratory of Urban Agriculture (North China), Beijing University of Agriculture, Beijing, China; ^3^ Institute of Wetlands, Chinese Academy of Forestry, Beijing, China; ^4^ Beijing Key Laboratory of Wetland Ecological Function and Restoration, Chinese Academy of Forestry, Beijing, China; ^5^ School of Ecology and Nature Conservation, Beijing Forestry University, Beijing, China

**Keywords:** wetland plant, resource heterogeneity, interspecific interaction, competitive hierarchy, root-to-shoot ratio

## Abstract

Nutrient heterogeneity in soil widely exists in nature and can have significant impacts on plant growth, biomass allocation, and competitive interactions. However, limited research has been done to investigate the interspecific competitive intensity between two clonal species in a heterogeneous habitat. Therefore, this greenhouse experiment was conducted with two clonal species, *Phragmites australis* and *Scirpus planiculumis*, exposed to heterogeneous and homogeneous patches of soil nutrients at five different planting ratios (0:4, 1:3, 2:2, 3:1 and 4:0), to assess the effects of both soil heterogeneity and interspecific competition on plant growth. It was found that soil nutrient heterogeneity significantly enhanced *P. australis*’ interspecific competitive capacity and biomass by promoting a 20% increase in belowground allocation. Interestingly, the planting ratio did not affect the magnitude of this net outcome. In contrast, the superior competitor *S. planiculumis* did not exhibit significant change of growth indicators to the heterogeneous soil patches. These findings imply that the uncertainties associated with human-induced redistribution of plant species may lead to a shift in dominance from other species to those like *P. australis*, which have strong nutrient foraging abilities in response to heterogeneity in emergent wetland plant communities.

## Introduction

Soils in all natural and managed environments exhibit spatial and temporal heterogeneity (Lechowicz and Bell, 1991; Day et al., 2003a; Mommer et al., 2012; Zhou et al., 2012; Costantini and Mocali, 2022). Clonal plants exhibit foraging responses to efficiently capture heterogeneous resources in soil, showing morphological plasticity or localized physiological changes in microsites with different nutrient levels (Hodge, 2004; Dong et al., 2015a; Dong et al., 2015b). In heterogeneous habitats, they tend to produce more shoots, ramets, and roots in high-nutrient microsites to search for high-quality patches. This enhanced clonal plant performance, measured by growth parameters, has been consistently observed in studies exploring the effects of soil nutrient heterogeneity (de Kroon, 2007; de Kroon et al., 2009; Sun et al., 2022). Previous research has also reported positive effects of soil nutrient heterogeneity on some individual plants (Wijesinghe and Handel, 1994; Roiloa and Retuerto, 2006; Bauerle et al., 2008; Peng et al., 2013) as well as plant populations (Day et al., 2003a; Xue et al., 2016).

Individual clonal plants have been found to have the capacity to concentrate their roots in nutrient-rich patches, leading to changes in interspecific competition (Robinson et al., 1999; Semchenko et al., 2007). This characteristic of clonal plants in interspecific interactions may be more advantageous than those of non-clonal species when the plants expand horizontally across a heterogeneous habitat (Robinson et al., 1999; Semchenko et al., 2007; Balestri et al., 2022). However, the effects of nutrient heterogeneity on interspecific interactions have mainly been explored using specific terrestrial plant species as experimental material, and few studies have examined this question in the context of wetland plant species, which are primarily distributed in the aquatic-terrestrial ecotone (Day et al., 2003b; Štěpán et al., 2004; Mommer et al., 2012). Furthermore, little is known about the possible complexity of interactions between two clonal species in such ecosystems, as empirical research on interspecific competitive intensity between two clonal species in a heterogeneous habitat is scarce (Mommer et al., 2012; Dong et al., 2015b). Consequently, the influence of soil nutrient heterogeneity on competition intensity and potentially community structure remains poorly understood.

Species responses to nutrient distribution in a competitive environment depend on the competitive strength of the neighbouring species (Mommer et al., 2012). Most tests of interspecific competitive intensity have focused on interactions between members of a single pair or a few pairs of species at a population density of 1:1 (e.g., Maestre and Cortina, 2004; Li et al., 2014; Paradis et al., 2014). However, population densities regularly change with various biotic or abiotic environmental factors (Stoll and Bergius, 2005). Therefore, the actual situation is extremely complicated, and neighbouring species growing in a heterogeneous environment may experience different interaction strengths that may partly depend on their relative abundances. These changes may result in a dynamic balance of inter- and intraspecific competition among individual plants. However, the effect of the plant relative abundance on competitive intensity has not been widely studied and not at all in heterogeneous environments (but see Čuda et al., 2015).

To address these research gaps, in this study, we aimed to understand the effects of soil nutrient heterogeneity and planting density ratio on the growth and interspecific interaction between two clonal plants. Specifically, we addressed the following questions: (1) Does nutrient heterogeneity influence the growth and competitive outcomes of clonal species? (2) How do the effects vary across different planting density ratios? To answer these questions, we conducted a greenhouse experiment in which two common emergent wetland clonal species growing at different planting densities were exposed to heterogeneous and homogeneous distributions of soil nutrients.

## Materials and methods

### Species and sampling

*Phragmites australis* (Cav.) Trin. ex Steud (Poaceae) and *Scirpus planiculumis* Fr. Schmidt (Cyperaceae) are the experimental species selected for this study. These species are perennial, herbaceous clonal plants that can reproduce from both seeds and clonal stems or tubers. They are capable of growing in a broad range of habitats, including semi-moist to wetland environments (Thevs et al., 2012; Peng et al., 2013).

*Phragmites australis*, a perennial, rhizomatous C3 grass, is known as a high nutrient specialist due to its increased growth and reproductive outputs in response to nutrient availability. This characteristic has allowed the species to become invasive in many areas around the world (Saltonstall and Stevenson, 2007; Kettenring et al., 2015; Long et al., 2017). On the other hand, *Scirpus planiculumis* is a rhizomatous, perennial emergent wetland plant. Both *P. australis* and *S. planiculumis* have asexual organs such as rhizomes and tubers that allow them to survive for several years and establish new populations through the formation of new shoots and rhizomes (Peng et al., 2013). These two species commonly co-occur in natural wetlands and share similar life history, root traits, and growth forms.

To conduct the experiment, we collected young *P. australis* and *S. planiculumis* seedlings of similar size (1.5 mm in length, 0.5 mm in width, and 0.4 mm in thickness) from a specific location in Beijing. Over 200 ramets per species were collected and acclimated to indoor conditions in a greenhouse for 10 days. For the experiment, a total of 120 thriving ramets of each species were selected, along with an additional 30 ramets for initial measurements. The initial average total dry mass was determined to be 2.34 ± 0.42 g for *P. australis* and 2.51 ± 0.36 g for *S. planiculumis*. These measurements were taken before transplanting the ramets.

### Experimental design

**Figure 1:**
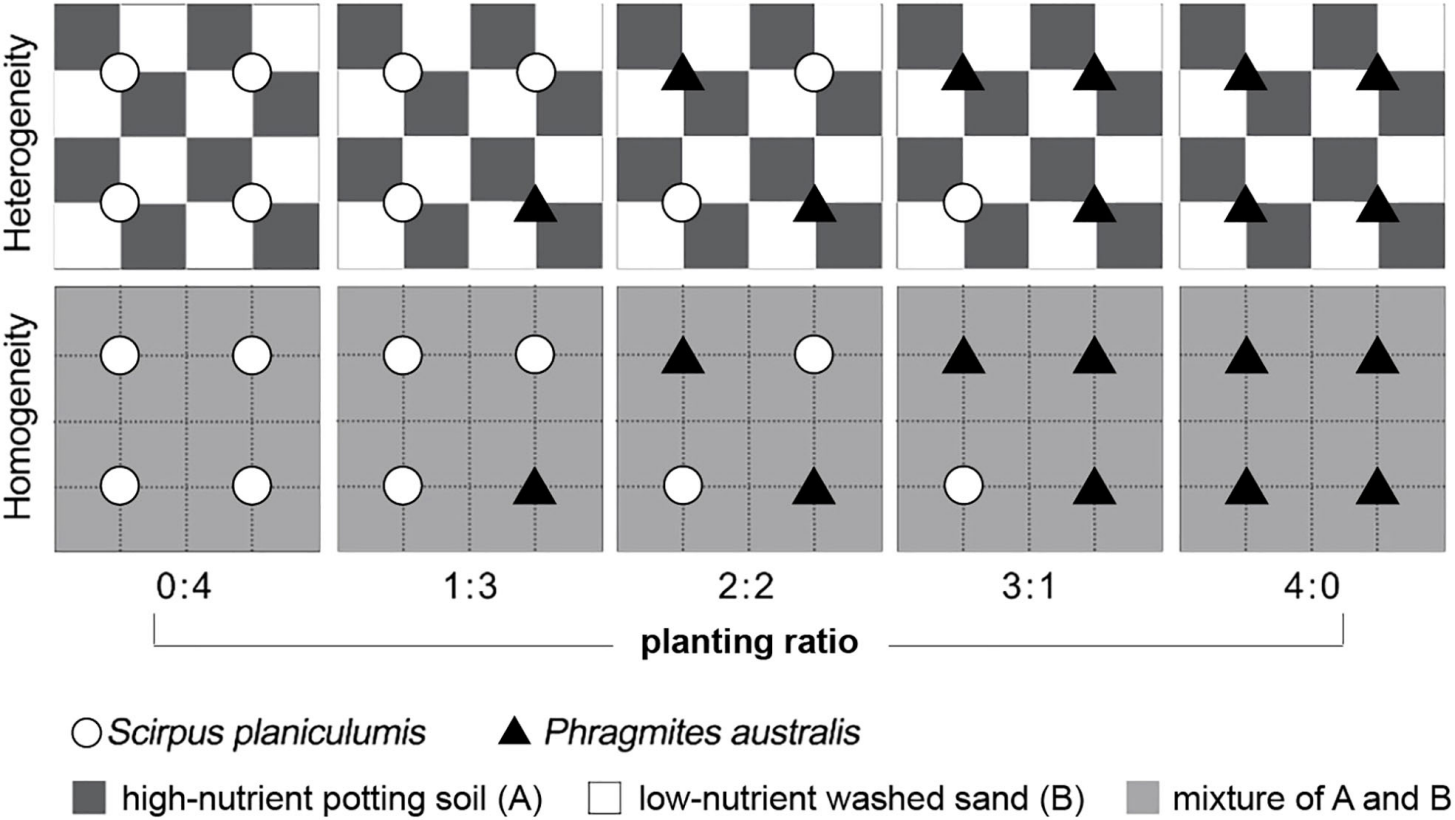
Schematic representation of the experimental design. Dark grey and white squares represent high-nutrient commercial potting soil **(A)** and low-nutrient washed sand **(B)** patches, respectively; light grey squares received the mean nutrient level between the high and low levels. The total amount of soil nutrients in a container was the same in all treatments. Open circles and filled triangles mark the positions where plants of *Scirpus planiculumis* and *Phragmites australis* were planted, respectively. There are six replicate containers (28 cm long × 28 cm wide × 20 cm deep) for each of the ten treatments and thus 60 plastic containers in total.

The experimental design consisted of ten treatments and involved two crossed factors: soil nutrient heterogeneity and planting ratio. There were six replicate containers for each of the ten treatments, resulting in a total of 60 plastic containers. The planting ratio ranged from 0:4 to 4:0 for *Phragmites australis* and *Scirpus planiculumis*, and the specific ratios are shown in **Figure 1**. For the heterogeneous soil treatment, alternating 8 × 8-cm patches in a container were filled with commercial potting soil, which had a high nutrient content. Alternate patches were filled with washed sand collected from the bank of an artificial lake, which had a low nutrient content. In the homogeneous soil treatment, each container was filled in the same way, and the soil was thoroughly mixed. The total amount of soil nutrients was identical in all communities.

On 14 April 2016, following the predetermined cultivation density ratios, four ramets of each species were transplanted in a vertical position 8 cm away from the corner to ensure equal access to high- and low-nutrient patches. In the homogeneous treatment, the plants were positioned similarly relative to each other (refer to **Figure 1**). Throughout the experiment, all containers were sprayed weekly with an insecticide dissolved in water. Additionally, tap water was added slowly to the containers to maintain the moisture of the soil surface. The mean temperature and mean relative humidity in the greenhouse during the pre-cultivation and experiment were 19.2°C and 72.8%, respectively (measured by iButton DS1923; Maxim Integrated Products, Sunnyvale, CA, USA).

### Data acquisition and analysis

After 10 weeks, on 23 June 2016, all *P. australis* and *S. planiculumis* plants were separately harvested. The plants were separated into above- and belowground parts, oven dried at 70°C for 72 h and weighed. For each species in each container, we measured the total length of the rhizomes and counted the number of ramets to represent the asexual reproductive performance. No plants flowered during the experiment. To describe the effects of soil nutrient heterogeneity on the interspecific interaction, we calculated two growth indices: the root-to-shoot ratio (R/S) and the relative growth rate (RGR) of the two emergent wetland species during the 10 weeks. The R/S was calculated as R/S = belowground biomass/aboveground biomass. The RGR was calculated based on the total dry biomass and initial biomass using the formula RGR = (lnTB_t_ – lnTB_0_)/t, where TB_t_ is the total biomass at time t, TB_0_ is the initial dry biomass, and t is the experimental duration (Wang et al., 2016). The duration time was 70 days (from 14 April to 23 June 2016). The initial dry biomass was calculated by combining the fresh biomass of each ramet with a constant average water content measured by an additional 30 ramets.

All growth data were separately analyzed for the two species using two-way ANOVA. The independent variables were soil nutrient heterogeneity (heterogeneous or homogeneous soil) and the planting ratio (the ratio of *P. australis* and *S. planiculumis* were 0:4, 1:3, 2:2, 3:1 and 4:0), while the dependent variables included total dry biomass, above- and belowground biomass, number of ramets, rhizome length, R/S, and RGR. Data on the total and aboveground biomasses of *S. planiculumis* were log transformed before analysis to meet the assumptions of homoscedasticity and normality. Differences between the heterogeneous and homogeneous soil nutrients within each treatment were tested using a paired t-test.

To quantitatively measure the intensity of interspecific interactions between the two emergent wetland species, we calculated the log response ratio (LnRR). The LnRR is widely used in ecology to quantify species interactions. The formula is LnRR = ln (B_with_/B_without_), where B_with_ and B_without_ represent the value of a growth variable in treatments with and without interspecific interactions, respectively. We calculated the LnRR based on total dry biomass and above- and belowground biomass. Smaller values of LnRR indicate higher interspecific competition intensities, with a more negative value indicating greater competition and a more positive value indicating a more facilitative interaction. We then used two-way ANOVA and paired t-tests to test the effects of soil nutrient heterogeneity and planting density ratio on the LnRR of both species. All statistical analyses were conducted in SPSS 20.0 (Statistical Product and Services Solutions, version 20.0; SPSS Inc., Chicago, IL, USA). Effects were considered significant at *P* < 0.05 (Dibble et al., 2013; Li et al., 2014; Zhou et al., 2017).

## Results

### Divergent responses of interspecific interaction to soil nutrient heterogeneity

**Figure 2:**
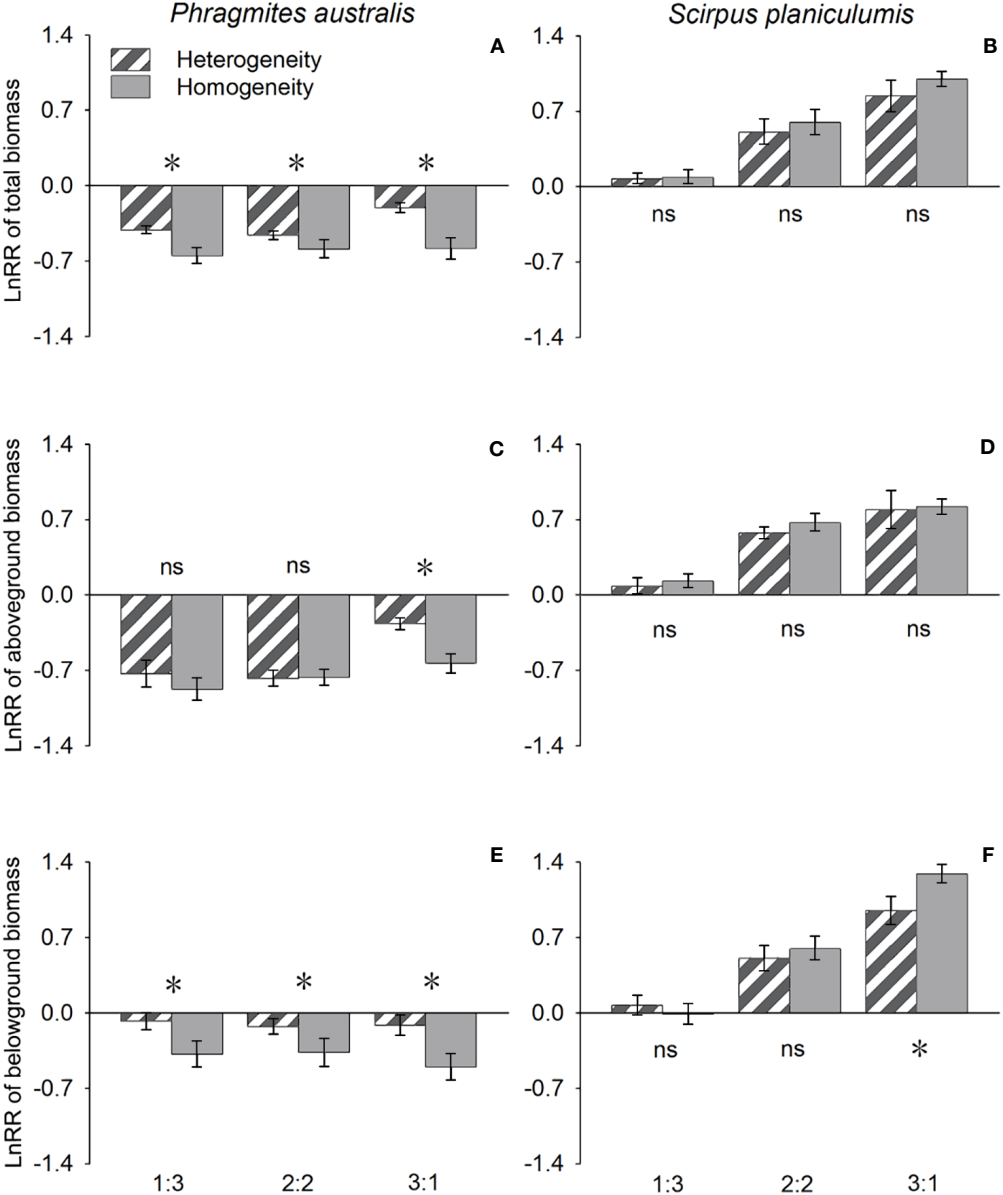
Effects of soil nutrient heterogeneity (heterogeneous and homogeneous soil) and planting density ratio (the proportions of *Phragmites australis* and *Scirpus planiculumis* were 1:3, 2:2 and 3:1) on the mean (± SE) log response ratio (LnRR) of **(A)** *Phragmites australis* based on the total biomass, **(B)** *Scirpus planiculumis* based on the total biomass, **(C)** *Phragmites australis* based on the aboveground biomass, **(D)** *Scirpus planiculumis* based on the aboveground biomass, **(E)** *Phragmites australis* based on the belowground biomass, and **(F)** *Scirpus planiculumis* based on the belowground biomass. A more negative value of the LnRR indicates greater competition intensity between two species, and a more positive value indicates a more facilitative interaction. Symbols (*) at the ends of the bars indicate that biomass significantly differed between the two soil nutrient heterogeneity treatments (paired t-test, P < 0.05); “ns”: *P* > 0.05. Means + SE are given. See **Table 1** for ANOVA results.

The effects of interspecific interaction were significantly influenced by the planting ratio, as indicated by the LnRR values in **Table 1**. Specifically, for *P. australis*, the LnRR values were consistently negative, suggesting that the interaction with *S. planiculumis* was competitive for all types of biomass (**Figure 2**). Furthermore, an increase in the planting ratio of *P. australis* resulted in a significant increase in LnRR values for total biomass and aboveground biomass, while the value for belowground biomass decreased (**Figure 2**). Interestingly, the competitive advantage of *P. australis* was observed to be greater in treatments with soil nutrient heterogeneity, as evidenced by higher LnRR values for total biomass and belowground biomass. However, for aboveground biomass, the difference in LnRR values between heterogeneous and homogeneous treatments was only significant at a planting ratio of 3:1, with no significant difference observed otherwise (**Figure 2**; **Table 1**).

**Table 1:** Results of two-way ANOVA testing the effects of soil nutrient heterogeneity and planting density ratio on the interaction intensity (LnRR) of *Phragmites australis* and *Scirpus planiculumis* based on total biomass, aboveground biomass and belowground biomass.

Variable	Heterogeneity (H)		Ratio (R)		H × R	
	*F*_1, 30_	*P*	*F*_2, 30_	*P*	*F*_2, 30_	*P*
Phragmites australis						
Total biomass	2.97	0.067	22.41	**<0.001**	1.86	0.173
Aboveground biomass	7.73	**0.009**	14.03	**<0.001**	3.32	**0.050**
Belowground biomass	13.76	**0.001**	0.32	0.727	0.25	0.782
Scirpus planiculumis						
Total biomass	0.99	0.328	32.42	**<0.001**	0.25	0.778
Aboveground biomass	0.47	0.500	25.53	**<0.001**	0.05	0.953
Belowground biomass	1.67	0.206	47.69	**<0.001**	1.82	0.180

Bold text indicates a significant difference (*P* < 0.05).

On the other hand, *S. planiculumis* exhibited mostly positive LnRR values, indicating that *S. planiculumis* benefited from the interaction with *P. australis*. Notably, a reduction in the planting ratio of *S. planiculumis* led to significantly increased LnRR values across all types of biomass, demonstrating the dependency of the interaction on the planting ratio (**Figure 2**). The soil nutrient heterogeneity treatment had no significant impact on LnRR values, except for belowground biomass at a planting ratio of 1:3 (**Figure 2**; **Table 1**).

### Heterogeneous soil nutrients affected the biomass allocation

**Figure 3:**
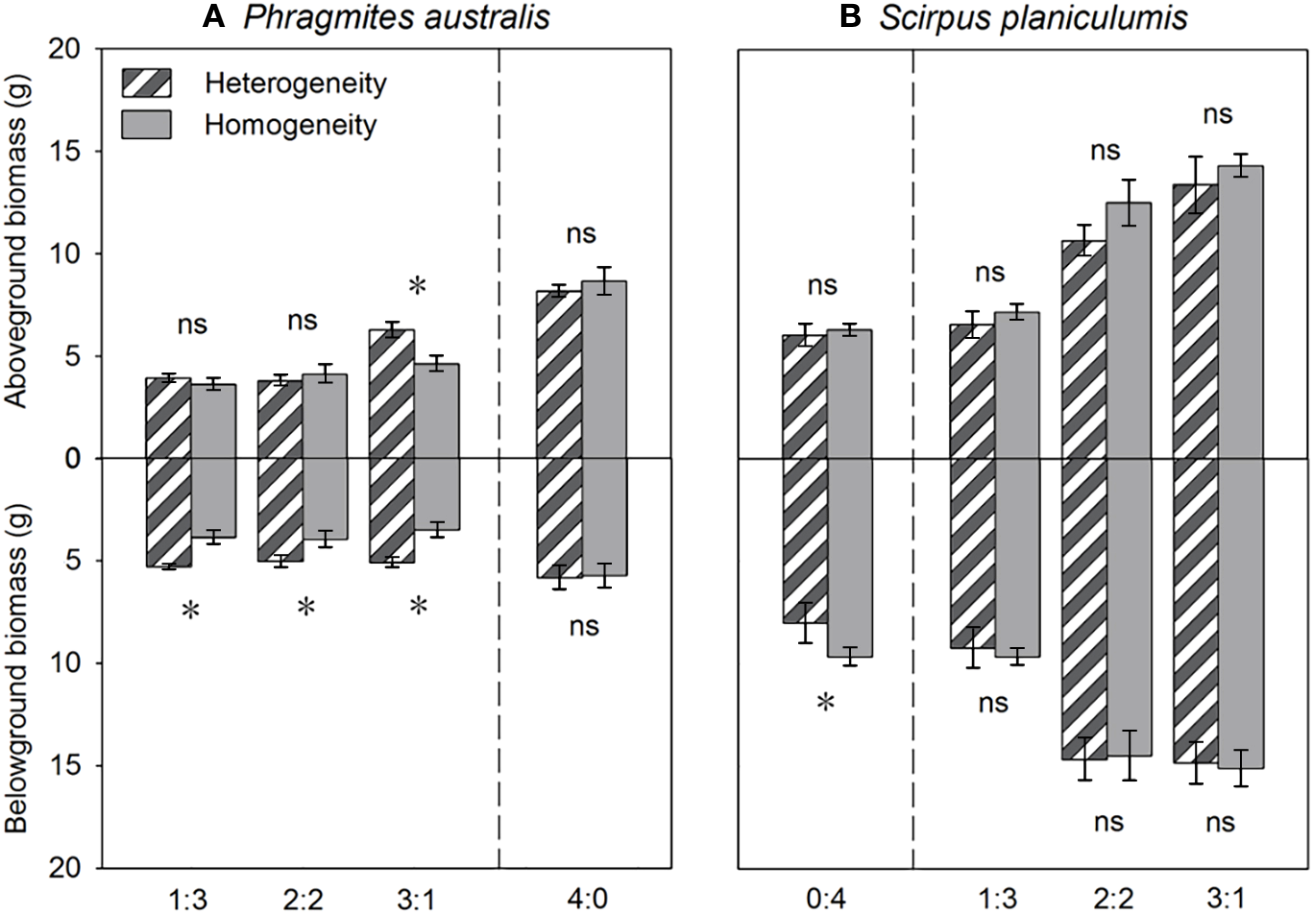
Effects of soil nutrient heterogeneity (heterogeneous and homogeneous soil) and planting density ratio (the proportions of *Phragmites australis* and *Scirpus planiculumis* were 0:4, 1:3, 2:2, 3:1 and 4:0) on the mean (± SE) above- and belowground biomasses of **(A)** *Phragmites australis* and **(B)** *Scirpus planiculumis*. The dashed line represents a separation between treatments with competition and without competition. Symbols (*) at the ends of the bars indicate that biomass significantly differed between the two soil nutrient heterogeneity treatments (paired t-test, *P* < 0.05); “ns”: *P* > 0.05. See **Table 2** for ANOVA results.

**Figure 4:**
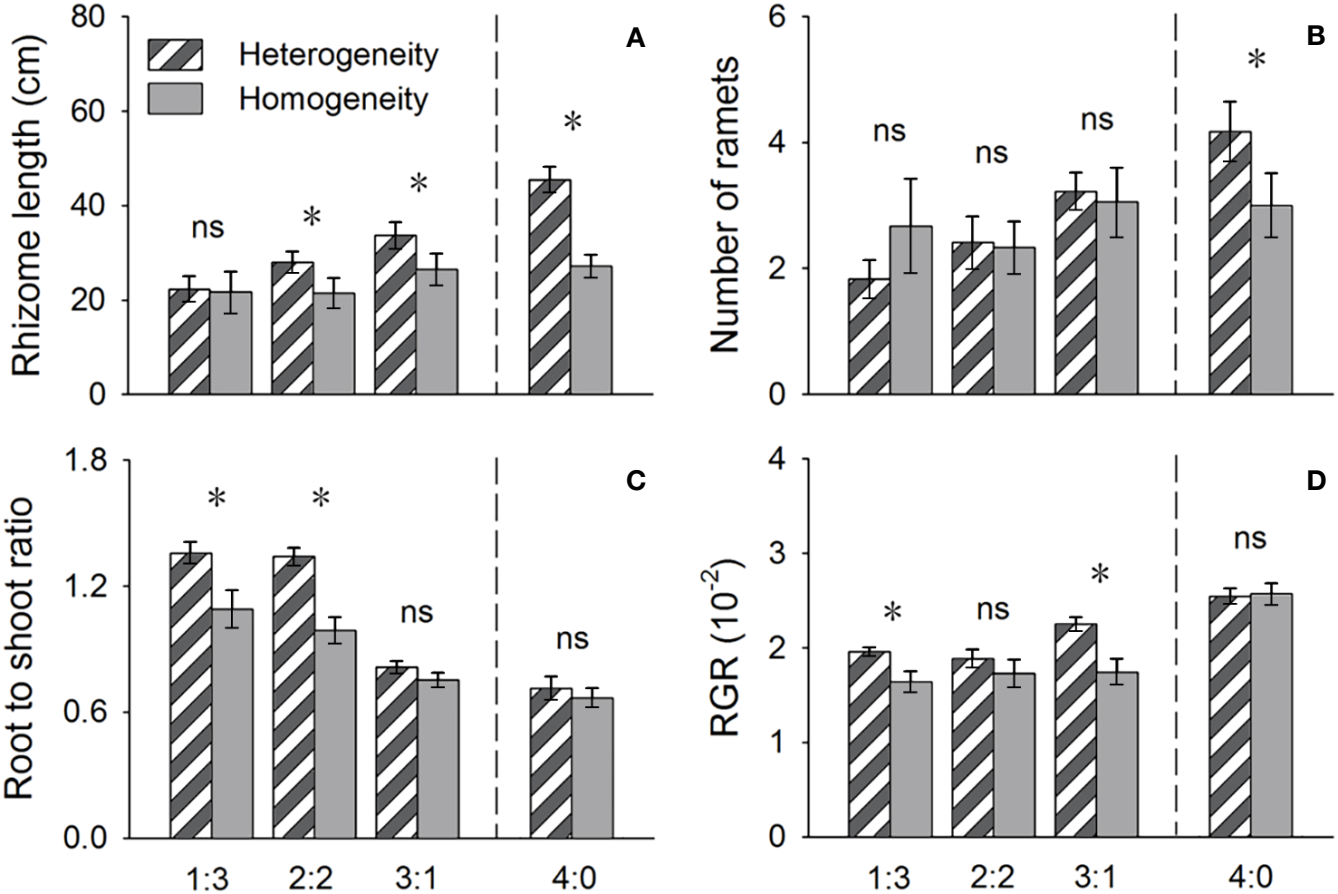
Effects of soil nutrient heterogeneity (heterogeneous and homogeneous soil) and planting density ratio (proportions of *Phragmites australis* and *Scirpus planiculumis* were 1:3, 2:2, 3:1 and 4:0) on the mean (± SE) **(A)** rhizome length, **(B)** number of ramets, **(C)** root to shoot ratio and **(D)** relative growth rate (RGR) of *Phragmites australis*. The dashed line represents a separation between treatments with competition and without competition. Symbols (*) at the ends of the bars indicate that the parameter significantly differed between the two soil nutrient heterogeneity treatments (paired t-test, P < 0.05); “ns”: *P* > 0.05. Means + SE are given. See **Table 2** for ANOVA results.

The total biomass, belowground biomass, rhizome length, and RGR were found to be significantly higher in heterogeneous treatments compared to homogeneous treatments (*P* < 0.05, **Figures 3**, **4**). Conversely, most growth measures of *S. planiculumis* exhibited a negative response to soil nutrient heterogeneity, either significantly (*P* < 0.05) or marginally (*P* < 0.10), except for the number of ramets (**Table 2**). Specifically, in homogeneous soil, *S. planiculumis* showed nearly 10% higher total biomass, particularly belowground biomass, compared to heterogeneous soil (*P* < 0.05, **Figure 3**).

**Table 2:** Results of two-way ANOVA testing the effects of soil nutrient heterogeneity and planting density ratio on the growth of *Phragmites australis* and *Scirpus planiculumis*.

Variable	Heterogeneity (H)		Ratio (R)		H × R	
	*F*_1, 40_	*P*	*F*_3, 40_	*P*	*F*_2, 40_	*P*
Phragmites australis						
Total biomass	6.55	**0.014**	27.29	**<0.001**	2.15	0.110
Aboveground biomass	1.01	0.320	53.23	**<0.001**	2.77	0.054
Belowground biomass	14.56	**<0.001**	6.08	**0.002**	1.43	0.248
Number of ramets	0.18	0.675	3.38	**0.027**	1.40	0.256
Rhizome length	13.73	**0.001**	8.25	**<0.001**	2.86	**0.049**
Root to shoot ratio	20.66	**<0.001**	44.55	**<0.001**	3.61	**0.021**
RGR	9.75	**0.003**	22.19	**<0.001**	2.28	0.094
Scirpus planiculumis						
Total biomass *	6.73	**0.013**	74.29	**<0.001**	0.55	0.649
Aboveground biomass *	3.09	0.086	48.08	**<0.001**	0.12	0.950
Belowground biomass	22.36	**<0.001**	118.28	**<0.001**	11.16	**<0.001**
Number of ramets	0.74	0.394	26.31	**<0.001**	0.38	0.766
Rhizome length	12.96	**0.001**	106.73	**<0.001**	2.77	0.054
Root to shoot ratio	3.14	0.084	13.48	**<0.001**	3.92	**0.015**
RGR	6.69	**0.013**	74.14	**<0.001**	0.56	0.647

* indicates that these data were natural logarithm-transformed to meet the requirements of homoscedasticity and normality. Bold text indicates a significant difference (*P* < 0.05).

**Figure 5:**
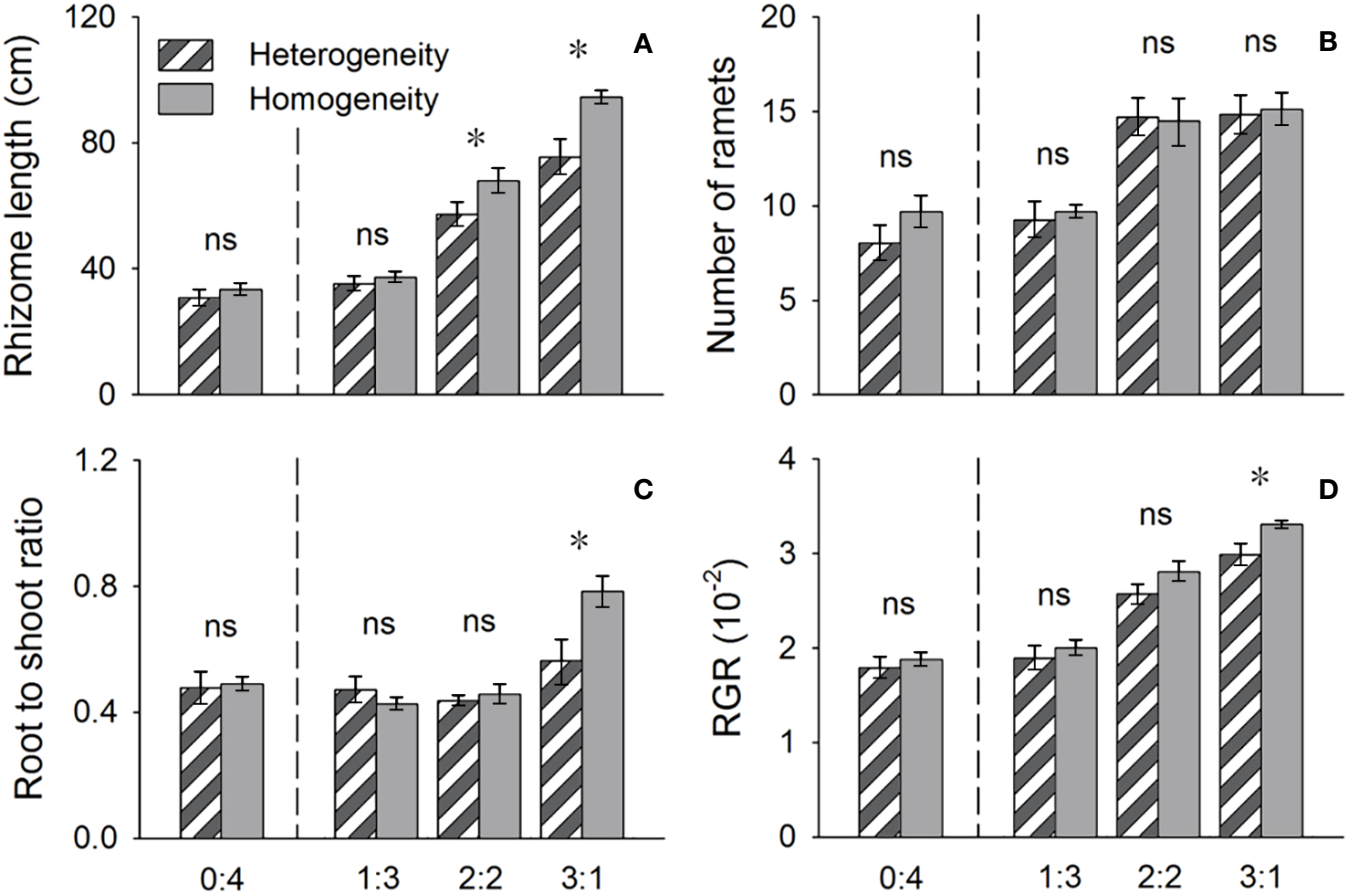
Effects of soil nutrient heterogeneity (heterogeneous and homogeneous soil) and planting density ratio (proportions of *Phragmites australis* and *Scirpus planiculumis* were 0:4, 1:3, 2:2 and 3:1) on the mean (± SE) **(A)** rhizome length, **(B)** number of ramets, **(C)** root to shoot ratio and **(D)** relative growth rate (RGR) of *Scirpus planiculumis*. The dashed line represents a separation between treatments with competition and without competition. Symbols (*) at the ends of the bars indicate that the parameter significantly differed between the two soil nutrient heterogeneity treatments (paired t-test, P < 0.05); “ns”: *P* > 0.05. Means + SE are given. See **Table 2** for ANOVA results.

The above- and belowground biomass allocation strategy of both species was influenced by soil nutrient heterogeneity and planting ratios. Under interspecific competition, *P. australis* allocated approximately 20% more biomass belowground than in homogeneous soil. However, this trend disappeared when the species was planted alone (4:0 planting ratio treatment; **Figure 3**). Similarly, the relative growth rate (RGR) and root to shoot ratio (R/S) of *P. australis* were significantly higher in heterogeneous soil treatments compared to homogenous treatments when competing with *S. planiculumis*. Interestingly, this promotion by nutrient heterogeneity was not observed when *P. australis* was planted alone (*P* < 0.05, **Figure 4**). For *S. planiculumis*, when planted alone, heterogeneous soils were associated with significantly lower belowground biomass compared to homogenous soils (*P* < 0.05, **Figure 3**). As the planting ratio of *S. planiculumis* decreased, there was no significant difference in biomass measures between heterogeneous and homogenous treatments (**Figure 3**). However, when interspecific competition was highest for *S. planiculumis* (3:1 planting ratio treatment), heterogeneous soil treatments resulted in significantly lower rhizome length, root to shoot ratios, and relative growth rate compared to homogenous soil treatments (*P* < 0.05, **Figure 5**).

## Discussion

### Effects of soil heterogeneity on individual plant growth

Our results suggested that soil nutrient heterogeneity increased the biomass of *P. australis*, primarily by increasing the belowground biomass. This species allocated more root biomass to nutrient-rich patches compared to nutrient-poor patches, benefiting from clonal integration and the division of labor (Day et al., 2003b; Roiloa et al., 2014; You et al., 2014; Zhang et al., 2016; Wang et al., 2022; Ma et al., 2023). However, *S. planiculumis* did not show an increase in biomass in heterogeneous soil, indicating that the effects of heterogeneity on clonal plant growth are context-specific (Kembel and Cahill, 2005). Our findings highlight that measures of plant growth alone may not sufficiently indicate the potential benefits of soil nutrient heterogeneity, as species differences exist in their response to heterogeneity (Johnson and Biondini, 2001; Liu and Dong, 2016).

Furthermore, the ability of roots to forage for nutrients partly depends on the spatial scale of soil heterogeneity (Day et al., 2003a; Hutchings and John, 2004). In our study, the 8 × 8-cm patches were more suitable for the root foraging of *P. australis* compared to *S. planiculumis*. This is potentially due to the low nutrient demand of *S. planiculumis*, which makes it insensitive to this scale of soil heterogeneity (Li et al., 2014). Additionally, our experimental design using natural substrate to create heterogeneous and homogeneous soil treatments may have overlooked accompanied changes in other soil abiotic and biotic features that could also influence plant growth strategy. To gain a more comprehensive understanding, future studies should monitor and analyze soil physicochemical and microbiological properties that potentially affect plant growth. Moreover, further research is needed to elucidate the effects of scale on the ecological responses of plant growth to soil heterogeneity.

### Effects of soil heterogeneity on interspecific interactions

In our study, the LnRR values for *P. australis* were all negative, indicating its competitive disadvantage in interactions with *S. planiculumis*. Conversely, the LnRR values for *S. planiculumis* were mostly positive, suggesting its competitive advantage over *P. australis* (**Figure 2**). When subjected to competition with neighbors, the interspecific competitive intensity for *P. australis* was greater in homogeneous soils compared to heterogeneous soils (the values were more negative in homogeneous soils). On the other hand, for *S. planiculumis*, the reverse pattern was observed (**Figure 2**). These findings align with previous studies that suggest patchy resource distributions can intensify interspecies competition (Day et al., 2003b; Roiloa et al., 2014; Wang et al., 2022). However, Mommer et al. (2012) also found that superior species were minimally affected by competition, while inferior species altered their growth strategy to produce more biomass in less favorable patches. Similarly, our study indicates that *S. planiculumis*, as the superior species, appeared to be insensitive to heterogeneous soil and interspecific interactions with neighbors. On the other hand, *P. australis*, the inferior species, adjusted its growth strategy by producing more roots in the heterogeneous environment. Cahill et al. (2010) also found comparable results for *Abutilon theophrasti*, where root responses to nutrient heterogeneity were only observed under competitive conditions. Despite methodological differences, our results, along with previous findings, suggest that root placement, influenced by environmental heterogeneity, heavily relies on interactions with competitive neighbors.

The competitive intensity for *P. australis* decreased with an increasing planting ratio of *P. australis* in the container (**Figure 2**). Both species exhibited enhanced growth, including greater biomass, rhizome length, and RGR, as *P. australis* density increased. These results indicate that *P. australis* was more affected by interspecific competition, while *S. planiculumis* was more impacted by intraspecific competition. This finding aligns with previous studies discovering that different species may experience different interaction strengths dependent on their relative abundances (Stoll and Bergius, 2005). The planting ratios designed in this study were likely to result in a dynamic balance of interspecific competition for *P. australis*, but intraspecific competition for *S. planiculumis.* Furthermore, these results remained consistent across different soil types (heterogeneous or homogeneous) in this study. However, the actual situation in the wild is more complicated as population densities regularly change with various biotic and abiotic environment factors, therefore, field studies are needed in the future to provide more details of competition for resources of different plant species in wild heterogeneous environments.

## Conclusions

Soil nutrient heterogeneity significantly increased the growth and interspecific competitive capacity of *P. australis* by driving the plants to allocate more biomass to belowground despite a competitive disadvantage with *S. planiculumis*. Comparatively, the superior competitor *S. planiculumis* showed no response to the heterogeneous soil patches. However, a reduction in the planting ratio of *S. planiculumis* in the community resulted in increased growth of *P. australis*. These results indicate that *P. australis* may have the capacity to be more efficient than other species in using heterogeneous resources. As environmental heterogeneity widely exists in natural ecosystems, uncertainties of redistribution of plant species induced by human activities may result in a shift in dominance from other species to those like *P. australis* with strong foraging abilities for nutrients in response to heterogeneity in emergent wetland plant communities, which needed to be given more attention in environmental monitoring. A full understanding of interspecific interactions between clonal plants requires the consideration of spatial heterogeneity in nutrient supply. Long-term studies at varying scales should be the focus of future research.

## Data availability statement

The raw data supporting the conclusions of this article will be made available by the authors, without undue reservation.

## Author contributions

JZ and LC conceived and designed the experiments. JZ, YJ, WL and JL executed the experiment and measured the data. JZ, YJ and JL analyzed the data and made the figures. LC, WL and YJ contributed to writing and editing the manuscript. JZ wrote the paper. ZM and YY contributed to the revision of the paper. All authors contributed to the article and approved the submitted version.
